# Adoptive cellular therapy prevents reconstitution of myeloid-derived suppressor cells in the glioma tumor microenvironment

**DOI:** 10.1093/noajnl/vdag054

**Published:** 2026-03-07

**Authors:** John W Figg, Caitland Love, Sofia Stansbury, Dan Jin, Connor Francis, Bayli DiVita Dean, Alexandra Reid, Mia Engelbart, Illeana West, Laura Falceto Font, Diana Feier, Ghaidaa Ebrahim, Rachael Bessey, David Hilferty, Oleg Yegorov, Changling Yang, Kaytora Long-James, Duane A Mitchell, Catherine T Flores

**Affiliations:** Preston A. Wells, Jr. Center for Brain Tumor Therapy, Lillian S. Wells Department of Neurosurgery, University of Florida, Gainesville, FL, USA; Preston A. Wells, Jr. Center for Brain Tumor Therapy, Lillian S. Wells Department of Neurosurgery, University of Florida, Gainesville, FL, USA; Preston A. Wells, Jr. Center for Brain Tumor Therapy, Lillian S. Wells Department of Neurosurgery, University of Florida, Gainesville, FL, USA; Preston A. Wells, Jr. Center for Brain Tumor Therapy, Lillian S. Wells Department of Neurosurgery, University of Florida, Gainesville, FL, USA; Preston A. Wells, Jr. Center for Brain Tumor Therapy, Lillian S. Wells Department of Neurosurgery, University of Florida, Gainesville, FL, USA; Preston A. Wells, Jr. Center for Brain Tumor Therapy, Lillian S. Wells Department of Neurosurgery, University of Florida, Gainesville, FL, USA; Preston A. Wells, Jr. Center for Brain Tumor Therapy, Lillian S. Wells Department of Neurosurgery, University of Florida, Gainesville, FL, USA; Preston A. Wells, Jr. Center for Brain Tumor Therapy, Lillian S. Wells Department of Neurosurgery, University of Florida, Gainesville, FL, USA; Preston A. Wells, Jr. Center for Brain Tumor Therapy, Lillian S. Wells Department of Neurosurgery, University of Florida, Gainesville, FL, USA; Preston A. Wells, Jr. Center for Brain Tumor Therapy, Lillian S. Wells Department of Neurosurgery, University of Florida, Gainesville, FL, USA; Preston A. Wells, Jr. Center for Brain Tumor Therapy, Lillian S. Wells Department of Neurosurgery, University of Florida, Gainesville, FL, USA; Preston A. Wells, Jr. Center for Brain Tumor Therapy, Lillian S. Wells Department of Neurosurgery, University of Florida, Gainesville, FL, USA; Preston A. Wells, Jr. Center for Brain Tumor Therapy, Lillian S. Wells Department of Neurosurgery, University of Florida, Gainesville, FL, USA; Preston A. Wells, Jr. Center for Brain Tumor Therapy, Lillian S. Wells Department of Neurosurgery, University of Florida, Gainesville, FL, USA; Preston A. Wells, Jr. Center for Brain Tumor Therapy, Lillian S. Wells Department of Neurosurgery, University of Florida, Gainesville, FL, USA; Preston A. Wells, Jr. Center for Brain Tumor Therapy, Lillian S. Wells Department of Neurosurgery, University of Florida, Gainesville, FL, USA; Preston A. Wells, Jr. Center for Brain Tumor Therapy, Lillian S. Wells Department of Neurosurgery, University of Florida, Gainesville, FL, USA; Preston A. Wells, Jr. Center for Brain Tumor Therapy, Lillian S. Wells Department of Neurosurgery, University of Florida, Gainesville, FL, USA; Preston A. Wells, Jr. Center for Brain Tumor Therapy, Lillian S. Wells Department of Neurosurgery, University of Florida, Gainesville, FL, USA

**Keywords:** adoptive immunotherapy, CCL12, glioblastoma, MDSC, migration

## Abstract

**Background:**

Glioblastoma (GBM) is an aggressive brain cancer infiltrated by immunosuppressive myeloid-derived suppressor cells (MDSCs) and confers poor prognosis. To address this, our group developed an adoptive cellular therapy platform specifically for primary central nervous system (CNS) malignancies that yielded significant survival benefits against multiple brain cancer models. Preclinically, this platform establishes proof-of-concept for lymphodepletion achieved through host conditioning with total body irradiation (TBI). While host conditioning is thought to remove immunosuppressive elements, the aim of this study was to determine how immune recovery is affected by adoptive cellular therapy.

**Methods:**

The adoptive cellular therapy platform includes myeloablative TBI, hematopoietic stem cell rescue, tumor-specific T cells, and dendritic cell vaccines. KR158B glioma-bearing mice were treated with adoptive cellular therapy and secondary lymphoid organs were evaluated using flow cytometry, spatial genomics, and multiplex protein analysis. Single-cell transcriptomics and trans-well migration assay evaluated the role of CCL12 on MDSC migration.

**Results:**

We show that adoptive cellular therapy allows for reconstitution of MDSC and tumor-associated macrophages in secondary lymphoid organs but prevents their accumulation in the tumor microenvironment (TME). This allows for the increased engraftment and activation of T cells within the TME. Next, we show that adoptive cellular therapy decreases CCL12 in the TME and neutralization of TAM-derived CCL12 in vitro inhibits MDSC migration in glioma.

**Conclusion:**

These findings suggest a previously unrecognized association between both loss of intratumoral immunosuppressive elements after immunotherapy and TAM-derived CCL12, a chemokine that promotes MDSC migration. Future in vivo studies will evaluate the causal role of CCL12 on MDSC recruitment in glioma.

Key Points

- Treatment with adoptive cellular therapy inhibits the accumulation of MDSCs and TAMs in glioma.
- This therapeutic approach enhances T-cell infiltration and activation within the glioma microenvironment.
- Neutralization of TAM-derived CCL12 limits MDSC migration in glioma in vitro.

Importance of the Study

Glioblastoma remains universally fatal despite treatment with surgery, radiotherapy, and chemotherapy, largely due to its profound immunosuppressive microenvironment. We demonstrate how an adoptive cellular therapy platform shapes immune recovery and identify a previously unrecognized mechanism regulating myeloid-derived suppressor cell (MDSC) migration in high-grade gliomas. By defining how myeloid cell reconstitution diverges in lymphoid organs and the tumor microenvironment, this work highlights barriers to effective immunotherapy and yields a potential strategy to overcome them. These findings carry direct translational significance, as this therapeutic platform is under active evaluation in phase 1 and 2 clinical trials.

Glioblastoma (GBM) is the most common primary malignant brain tumor diagnosed in adult patients.^1-3^ Despite multimodal management, patients diagnosed with GBM have a median overall survival (OS) of 15 to 18 months.^1-4^ Data from The Cancer Genome Atlas (TCGA) identified the immunosuppressive tumor microenvironment (TME) as a prognostic characteristic of GBM.^5^ The TME consists of tumor-associated macrophages (TAMs) and microglia, regulatory T and B cells, and myeloid-derived suppressor cells (MDSCs).^6-13^ It has also been observed that GBM patients have an enrichment of MDSCs in peripheral blood, and significantly higher frequencies of MDSCs within the tumor and peripheral blood relative to other cancers.^5-11^ In GBM patient samples, the intratumoral MDSC subpopulations are transcriptionally distinct, upregulating hypoxia and cell stress pathways and correlate with poor prognosis.^14^ Consequently, the immune regulatory role of MDSCs is a major barrier to therapeutic efforts against GBM.

To improve clinical outcomes for GBM patients, immunotherapeutic protocols including T-cell-based therapies have emerged as potential treatment options that synergize with current standards of care. Our group developed an adoptive cellular therapy (ACT) platform that simultaneously targets a plurality of antigens with a single treatment.^15-18^ In the murine system, this ACT platform employs bone marrow–derived dendritic cells (BMDCs) pulsed with total tumor RNA to activate and expand tumor-reactive T cells in vitro. After host conditioning using total body irradiation (TBI), hematopoietic stem cells (HSCs) and T cells are adoptively transferred into the glioma-bearing host. Adoptive cellular therapy resulted in a significant survival benefit in multiple preclinical models of central nervous system (CNS) malignancies, including high-grade gliomas (HGGs) such as KR158B and GL261, group 3 medulloblastoma (NSC MB), and sonic hedgehog (*Ptch+/−*), and this finding has been translated to phase I & II clinical trials (FDA IND #17298, 14058, 23881).^15-21^ Previous mechanistic studies revealed that ACT leads to significant decreases in the accumulation of endogenous MDSCs in the TME while mediating anti-tumor T-cell infiltration and activation in the tumor site.^15-20 ^ The efficacy of this platform requires lymphodepletive host conditioning.^15^ For proof-of-principle in preclinical models, host conditioning in these studies was achieved using TBI, either through a lymphodepletive dose (5 Gy) or through a myeloablative dose (9 Gy); however, the differences in lymphodepletive and myeloablative host conditioning regimens on immune cell reconstitution in the periphery and in the TME have not yet been characterized until this study.

Here, we investigated immune reconstitution in glioma-bearing mice after host conditioning, HSCs, and ACT. We found myeloablation and HSCs to profoundly reduce TAM and MDSC frequencies within the TME. Myeloablation, HSCs, and ACT led to robust immune reconstitution in secondary lymphoid organs, and increased tumor-infiltrating T cells were in the TME. Crucially, ACT prevented reconstitution of MDSCs and TAMs at the tumor site, leading us to believe that the overall immunosuppression in the TME is appreciably reduced after therapy. We further demonstrate that ACT decreases CCL12 in the TME and that in vitro neutralization of macrophage-derived CCL12 abrogates MDSC migration. These findings underscore implications for enhancing ACT as an effective treatment strategy for HGGs.

## Methods

### Ethics

All studies were approved by the University of Florida Institutional Animal Care and Use Committee (IACUC 202100000053 and 202200000409).

### Mice

Female C57BL/6J mice (Jackson Laboratories, strain #00664) or C57BL/6-Tg (UBC-GFP)30Scha/J (GFP) mice aged 5 to 8 weeks were used for experiments. All investigators adhered to the “Guide for the Care and Use of Laboratory Animals” under UF IACUC 202100000053 and 202200000409.

### RNA Isolation

Total tumor RNA isolation from the KR158B cell line was performed with RNeasy mini kit (Qiagen, cat. 74104) per the manufacturer’s protocol.

### MDSC Cell Culture

Bone marrow from the tibia and femur were harvested from female C57BL/6J mice aged 6 to 8 weeks. MDSC induction was adapted from previously published work.^22^

### Brain Tumor Model Cell Culture

KR158B glioma cells were cultured in Dulbecco Modified Eagle Medium (DMEM, Thermo Fisher, cat. 11-965-118) without sodium pyruvate and supplemented with 1% penicillin-streptomycin (Fisher Scientific, cat. 15140163) and 10% fetal bovine serum (FBS, Thermo Fisher Scientific, cat. A5256801). Cells were grown in a humidified incubator at 37°C with 5% CO_2_.

### Orthotopic Brain Tumor Model

Tumor-bearing experiments were performed in syngeneic female C57BL/6J mice. A total of 10^4^ KR158B or GL261 cells were implanted as previously described.^23^^,^^24^

### Tumor-Reactive T Cells for ACT

Bone marrow from the tibia and femur was harvested from female mice aged 6 to 9 weeks. Isolated cells were cultured in RPMI-1640 buffer (Thermo Fisher, cat no. 11875093) supplemented with 5% FBS, 1% penicillin-streptomycin, 1% HEPES, 1% NEAA, 1% sodium pyruvate, 0.5% L-Glutamine, 0.1% BME, 10 ng/mL GM-CSF (R&D, cat. 415-ML/CF), and 10 ng/mL IL-4 (R&D, cat. 404-ML/CF) for 9 days. Dendritic cells (DCs) were then electroporated with 25 μg RNA isolated from tumor tissue. Naïve mice were primed with 2.5 × 10^5^ DCs. After 1 week, splenocytes were then harvested and co-cultured ex vivo using DCs and IL-2 (50 U/mL, R&D, cat. 402-ML/CF) for 5 days. A total of 10^7^ total tumor RNA T cells were intravenously administered for ACT.

### Adoptive Cellular Therapy

The treatment of glioma-bearing mice began with 5-Gy lymphodepletion or 9-Gy myeloablation on day 4 postintracranial injection with X-ray irradiation (X-RAD 320, PXINC). To isolate HSCs, fresh bone marrow from the tibia and femur was harvested from female C57BL/6J mice aged 6 to 8 weeks. Lineage-negative HSCs are isolated using the lineage depletion kit (Miltenyi Biotec, cat. 130-090-858) to deplete CD5, CD45R (B220), CD11b, Gr-1 (Ly-6G/C), 7-4, and Ter-119-positive cells per manufacturer’s protocol. On day 5 postintracranial tumor injection, mice received a single intravenous injection with 10^7^ autologous ex vivo expanded total tumor RNA T cells with 5 × 10^4^ lineage-depleted HSCs. Beginning day 7 post-tumor injection, 2.5 × 10^5^ total tumor RNA-pulsed DCs were injected as previously described.^15^^,^^16^

### Brain Tumor Digestion

Brain tumors were mechanically dissociated with sterilized scissors and chemically digested with a mix consisting of 1 mL 10× hyaluronidase (Stem Cell Technologies, cat. 07462), 1 mL 10× Collagenase (Sigma-Aldrich, cat. C5138-500MG), 100 μL 100× DNase (Qiagen, cat. 79254), and 8 mL DMEM without sodium pyruvate and FBS. Sample mixtures were added into gentleMACS C tubes (Miltenyi Biotec, cat. 130-093-237), and placed onto the GentleMACS Octo Dissociator (Miltenyi Biotec, cat. 130-134-029) using the 37C-mTDK_2 program. Cell pellets are resuspended using 3100 μL of cold 1× PBS and 900 μL of cold debris removal solution (Miltenyi Biotec, cat. 130-109-989) and transferred to 15 mL polypropylene conical tubes (Fisher Scientific, cat. 14-959-49D). A total of 4 mL of cold 1× PBS was then overlayed with this mixture and centrifuged (4°C, 3000 × *g*, 10 minutes, brakes and acceleration set to 2). After aspirating the buffy coat, the pellet was then used for downstream assays.

### Protein Quantification

Protein quantification was performed using a bicinchoninic acid assay (Pierce BCA Protein Assay Kit, Thermo Fisher, cat. 23227) per the manufacturer’s protocol.

### Tumor Lysate Generation

Tumors were excised as previously described in the “Brain Tumor Digestion” section and snap-frozen using liquid nitrogen. Frozen tissue was crushed using a mortar and pestle to generate a fine powder and mixed with 1 mL of RIPA lysis buffer (Sigma-Aldrich, cat. R0278-50mL) containing 100× HALT protease inhibitor cocktail (Thermo Fisher, cat. 78440). Samples were incubated on ice for 30 minutes. Then, samples underwent centrifugation (4°C, 14 000 × *g*, 15 minutes). Supernatants were removed and stored in liquid nitrogen.

### CCL12 Enzyme-Linked Immunosorbent Assay

CCL12 protein quantification was performed using a CCL12 ELISA (Thermo Fisher Scientific, cat. EMCCL12) per the manufacturer’s protocol.

### In Vitro Cell Migration

Induced MDSCs were generated as described previously in the “MDSC Cell Culture” section.^22^ Cells were diluted to a final concentration of 2,000 cells/µL in migration buffer consisting of RPMI-1640, 25 mM HEPES, 1% penicillin-streptomycin, and 0.1% BSA (>98% quality). In vitro migration was assessed using a trans-well-96-well plate with 5 μm polycarbonate membrane (Corning, cat. 3388). Recombinant mouse CCL2 (R&D Systems, cat. 479-JE) and CCL12 (R&D Systems, 428-P5) were diluted in the migration buffer and plated at 150 µL/well in the lower Boyden chamber. To validate the chemotaxis effect, chemokine was also plated with neutralizing antibody (αCCL2, R&D Systems, cat. AB-479-NA; αCCL12, R&D Systems, cat. MAB428-SP). Cells were plated at 150 µL/well in the upper Boyden chamber and incubated at 37°C in 5% CO_2_ for 6 hours. After incubation, the membrane was lightly shaken to remove migrated cells and then discarded. Migrated cells were analyzed using single color compensation on the BD FACS Symphony A3. Gating strategy is described in the “Flow Cytometry” section.

For analysis of migration into GCM (see the “MDSC Cell Culture” section) with neutralizing antibodies against CCL12 and/or CCL2 as previously described, GCM was plated at 150 μL/well in the lower Boyden chamber at equivalent protein concentration. Polyclonal goat IgG antibodies (anti-CCL2, anti-CCL12, and normal goat IgG control) were added in equivalent concentrations (low dose, 1 µg/mL; high dose, 10 µg/mL) to the lower Boyden chamber and incubated for 60 minutes with the GCM. MDSCs were plated in the upper Boyden chamber and plated at the same concentration and volume as previously described. Migrated cells were analyzed by flow cytometry.

### Flow Cytometry

Single-cell suspensions were prepared from tissues as described above and diluted to 1 x 10^6^ cells/100 μL FACS buffer. Subsequently, cells were washed with ice-cold PBS and stained with a viability dye for 10 minutes at 4°C in the dark. Subsequently, samples were stained for extracellular markers for 30 minutes at 4°C in the dark (Supplementary Table S1). Then, samples were fixed using 2% paraformaldehyde (Fischer Scientific, cat. AAJ19943K2) for 30 minutes at 4°C in the dark. Murine MDSC phenotypes used for gating were derived from Bronte et al.^25^ Representative gating strategy for immune populations is described in Supplementary Figure S1.

*Bromodeoxyuridine incorporation*: After staining extracellular markers, samples were fixed using fix/permeability buffer (Thermo Fisher, cat. 00-5521-00) for 60 minutes at 4°C in the dark. Following fixation, samples were washed twice using 1 mL FACS buffer. Next, samples are incubated with DNase I (Sigma, cat. D4513-1VL) working solution for 1 hour at 37°C in the dark. Samples were washed twice with permeabilization buffer. Next, samples were stained using fluorescence-activated cell antibodies against intracellular markers (see Supplementary Table S1). Samples were incubated for 60 minutes at 4°C in the dark. After incubation, samples were washed with 1 mL permeabilization buffer, centrifuged (4°C, 500 × *g*, 5 minutes), and stored in 4°C until acquisition via flow cytometry.

### Digital Spatial Profiling

C57BL6/J mice were orthotopically implanted with KR158B cells and subsequently treated with irradiation, HSCs derived from C57BL/6-Tg (UBC-GFP)30Scha/J (GFP) mice, and ACT. Histological slides of brain sections were sent to be stained for CD45, CD3, GFP (as a marker of HSC-derived cells) and nuclei, and processed following nanoString GeoMx Digital Spatial Profiling (DSP) for whole transcriptomic analysis. The stained slides were loaded onto the profiler, and 24 ROIs (200 nuclei each) were chosen within the tumor on each sample. Genes were selected based on previous studies focused on macrophage, MDSC, and T-cell gene sets.^26-33^

### Multiplex Cytokine/Chemokine Analysis

KR158B glioma cells were implanted into female C57BL/6J mice and were treated with ACT as previously described. After 28 days postimplantation, tumors were excised, and lysates were generated as previously described in the “Tumor Lysate Generation” section. The concentration of each cytokine/chemokine per sample was normalized to total protein. Samples were analyzed using the Mouse Cytokine/Chemokine 44-plex Discovery Assay Array (Eve Technologies, cat. MD44).

### Survival Analysis

The complete human GBM patient dataset was mined from GEPIA2 to extract gene expression and clinical parameters.^34^ This platform was accessed on May 5, 2025. Patients were stratified into median *Ccl2*, *Cd14*, and *Fcgr3a* expression categories (low, <50th percentile; high, >50th percentile). Descriptive statistics were generated through R analysis software, depicting *P* values for the log-rank and hazard ratio for patients stratified into the high expression group. Group sizes, denoted by “n,” are also reported. Survival curve comparisons were calculated using log-rank (Mantel-Cox test) and graphically illustrated through R Graphics Output.

### Statistical Analysis

Statistical analyses, including log-rank (Mantel-Cox test to compare Kaplan-Meier survival curves), *t* tests (2 group comparisons), 1-way ANOVA (>2 group comparisons), 2-way ANOVA, and graphical depictions, were generated in GraphPad Prism version 10.3.0 to determine statistically significant differences between groups. A normal distribution is observed in the data with variance similar between groups statistically compared. Statistical significance is determined at a *P* value of <.05. For animal studies where tissues were analyzed for biological endpoints, an n of 5 to 6 mice per group is used. For animal studies where survival was analyzed, group sizes harbored an n of ≥7. Authors preestablished that no animals or samples were excluded from analysis. Randomization of mice occurred on postimplantation day 1 prior to administering any treatment or irradiation.

## Results

### Myeloid Cell Reconstitution in Gliomas Is Suppressed After Myeloablation

**Figure 1.**
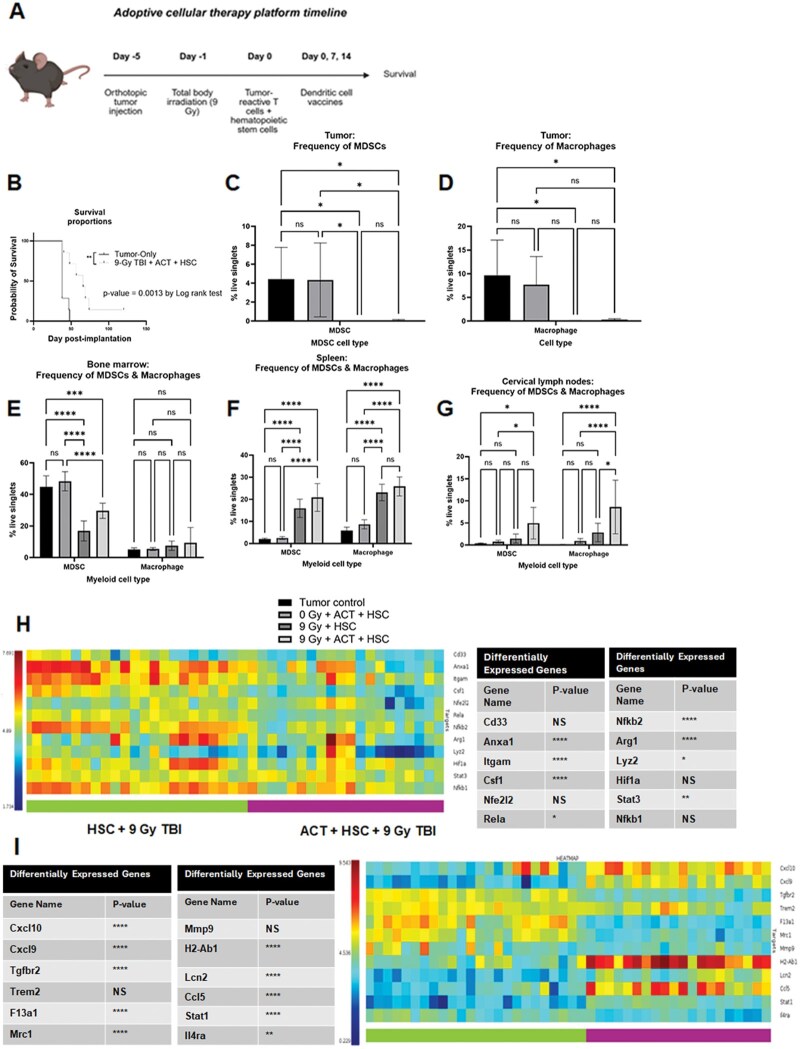
Characterization of myeloid cell populations in lymphoid organs and tumor microenvironment following myeloablation, HSCs, and adoptive cellular therapy. (A) Schematic of treatment groups receiving adoptive cellular therapy with TBI (created with biorender.com). Bone marrow, cervical lymph nodes, spleen, and tumor were harvested 1-week post-treatment for flow cytometric analysis. (B) Kaplan-Meier survival curve comparing adoptive cellular therapy–treated mice with untreated tumor controls. Statistical analysis was performed using the log-rank test. (C) Reconstitution of granulocytic, monocytic myeloid-derived suppressor cells and (D) macrophages in tumor across treatment groups. Statistical analysis was performed using the 2-way ANOVA test. (E) Reconstitution of granulocytic, monocytic myeloid-derived suppressor cells, and macrophages in bone marrow across treatment groups. Statistical analysis was performed using the 2-way ANOVA test. (F) Reconstitution of granulocytic and monocytic MDSCs and macrophages in spleen across treatment groups. Statistical analysis was performed using the 2-way ANOVA test. (F) Reconstitution of granulocytic and monocytic MDSCs in the glioma microenvironment across treatment groups. Statistical analysis was performed using the 2-way ANOVA test. (G) Reconstitution of granulocytic and monocytic MDSCs and macrophages in cervical lymph nodes across treatment groups. Statistical analysis was performed using the 2-way ANOVA test. (H) Heatmap of RNA expression of MDSC-associated genes between 9 Gy myeloablative TBI and HSCs and 9 Gy myeloablative TBI, HSCs, and adoptive cellular therapy. (I) Heatmap of RNA expression of M1 and M2 macrophage associated genes between 9 Gy myeloablative TBI and HSCs and 9 Gy myeloablative TBI, HSCs, and adoptive cellular therapy. Statistical analysis was performed using the 2-way ANOVA test. *P ≤ .05. ** *P* ≤ .01. ***P ≤ .001. ****P ≤ .0001; error bars: mean ± standard deviation. Abbreviations: ANOVA, analysis of variance; HSCs, hematopoietic stem cells; MDSCs, myeloid-derived suppressor cells; TBI, total body irradiation.

Previously, our group determined that ACT significantly extended overall survival across models of HGG, brain stem glioma, and medulloblastoma (Figure 1A and B).^15-17^^,^^19^^,^^20^ Mechanistic evaluation determined that adoptive cellular therapy displaced host-derived MDSCs from the TME.^16^ We first conducted studies to measure the impact of 9-Gy myeloablative host conditioning after ACT on myeloid cell reconstitution. To do this, KR158B glioma-bearing mice were randomized into groups that received no treatment (tumor control), HSCs and ACT (0 Gy + ACT + HSC), myeloablative irradiation and HSCs (9 Gy + HSC), or myeloablative irradiation, HSCs, and ACT (9 Gy + ACT + HSC). Twenty-eight days after ACT, cells from the tumor, bone marrow, cervical lymph nodes, and spleens were isolated and processed for flow cytometry (Figure 1C-G). *Glioma*: Surprisingly, MDSC and TAM reconstitution was significantly decreased after myeloablation with HSCs and myeloablation with HSCs and ACT (Figure 1C and D). *Bone marrow:* We observed that MDSCs were significantly decreased in all groups receiving myeloablation and HSCs or myeloablation with HSCs and ACT (Figure 1E). We also observed macrophage reconstitution in bone marrow was not significantly different after myeloablation. *Spleen:* We observed that MDSC and macrophage reconstitution was significantly increased in groups that received myeloablation and HSCs or myeloablation, HSCs, and ACT relative to mice that received no myeloablation (Figure 1F). *Cervical lymph nodes:* We observed a significant increase in MDSCs and macrophages in mice treated with myeloablation with HSCs and ACT compared with mice that did not receive myeloablation. Additionally, we observed an increase in cervical lymph node-derived macrophages in mice treated with myeloablation and HSCs relative to those treated with myeloablation, HSCs, and ACT (Figure 1G). These results suggest that while lymphoid organs undergo myeloid immune reconstitution after myeloablation and ACT, there is a failure of myeloid TME reconstitution after myeloablation.

To validate our above findings, we next evaluated if transcriptional signatures associated with myeloid cell reconstitution were altered after myeloablation and ACT in the TME. DSP with GeoMx was performed on KR158B glioma either with or without ACT. Whole transcriptomic analysis revealed that gene expression of multiple MDSC-associated genes was significantly decreased after myeloablation with HSCs and ACT relative to myeloablation and HSCs, including *Anxa1*, *Itgam*, *Csf1*, *Rela*, *Nfkb2*, *Arg1*, *Lyz2*, *Stat3*, and *Nfkb1* (Figure 1H). Given the abundance of macrophages within the TME, we evaluated gene expression associated with M1-polarized and M2-polarized macrophages after treatment. We identified that transcriptional programs associated with M1-polarized macrophages were significantly increased after myeloablation with HSCs and ACT relative to myeloablation and HSCs, such as *Cxcl10*, *Cxcl9*, *H2-Ab1*, *Lcn2*, *Ccl5,* and *Stat1* (Figure 1I). Genes linked to M2-macrophages, including *Tgfbr2*, *F13a1*, *Mrc1* and *Il4ra*, showed significantly reduced expression following myeloablation with HSCs and ACT relative to myeloablation and HSCs (Figure 1I). Collectively, myeloablation alone significantly decreased myeloid cell reconstitution in the TME and the combination of myeloablation and ACT shifted myeloid transcriptional programs away from immunosuppression toward pro-inflammatory signatures.

To compare the effect of non-myeloablative and myeloablative TBI on immune cell reconstitution, we measured MDSCs and macrophages in groups that received 5-Gy lymphodepletive or 9-Gy myeloablative irradiation with or without ACT. *Bone marrow*: PMN-MDSC reconstitution was significantly decreased in bone marrow after 9-Gy myeloablation, HSCs, and ACT compared with 5-Gy lymphodepletion, HSCs, and ACT (Supplementary Figure S3A). *Spleen*: M-MDSC reconstitution was significantly increased after 9-Gy myeloablation and HSCs or 9-Gy myeloablation with HSCs and ACT compared with 5-Gy alone or 5-Gy with HSCs and ACT. PMN-MDSC and macrophage frequencies significantly increased in mice treated with 9-Gy myeloablation with HSCs and ACT relative to tumor controls, 5-Gy lymphodepletion monotherapy, and 5-Gy lymphodepletion with HSCs and ACT (Supplementary Figure S3B-D). *Glioma*: PMN-MDSC and macrophage reconstitution was significantly decreased after 9-Gy myeloablation and HSCs or 9-Gy myeloablation with HSCs and ACT compared with 5-Gy lymphodepletion, HSCs, and ACT (Supplementary Figure S3E and F). *Cervical lymph nodes*: Macrophage reconstitution was significantly increased after 9-Gy myeloablation with HSCs and ACT compared with 5-Gy lymphodepletion, HSCs, and ACT (Supplementary Figure S3G). Quantitative comparisons of log2-fold change in gene expression of MDSC and macrophage genes between 9-Gy myeloablation and HSCs and 9-Gy myeloablation, HSCs, and ACT using the above-mentioned spatial profiling is expressed in Supplementary Figure S3I and H. We also characterized myeloid cell proliferation, apoptosis and cell death after ACT (Supplementary Figure S5A-H).

### Significant T-Cell Infiltration After ACT and Myeloablation

**Figure 2.**
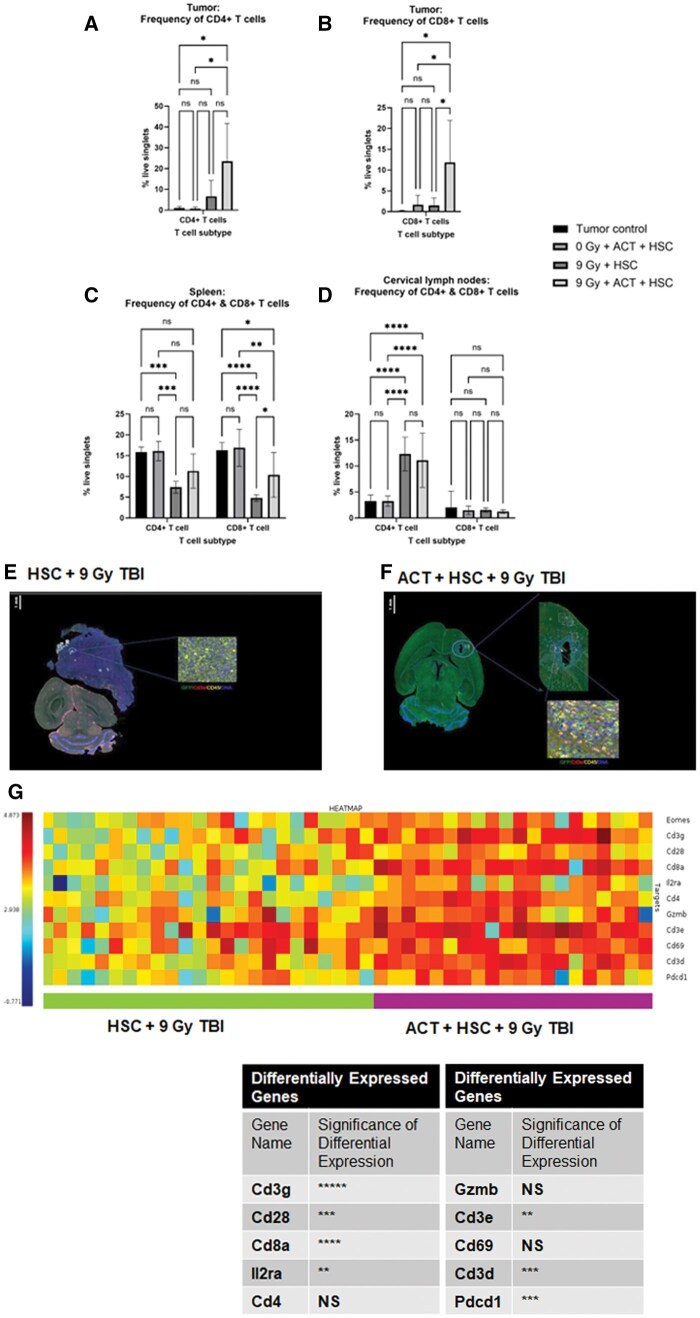
Characterization of lymphoid reconstitution and transcriptional programs in secondary lymphoid organs and tumor microenvironment after myeloablation, HSCs, and adoptive cellular therapy in glioma. (A) Reconstitution of CD4+ T cells in the tumor after treatment. Statistical analysis was performed using the 2-way ANOVA test. (B) Reconstitution of CD8+ T cells in the tumors after treatment. Statistical analysis was performed using the 2-way ANOVA test. (C) Reconstitution of CD4+ and CD8+ T cells in the spleen after treatment. Statistical analysis was performed using the 1-way ANOVA test. (D) Reconstitution of CD8+ T cells in the cervical lymph node after treatment groups. Statistical analysis was performed using the 1-way ANOVA test. (E and F) Spatial transcriptomics (GeoMx) was performed on formalin-fixed brain sections from mice bearing orthotopic KR158B gliomas that were either treated with (E) 9 Gy myeloablative radiation and HSCs or with (F) 9 Gy radiation, HSCs, and adoptive cellular therapy. Sections were stained for CD3 (red), CD45 (yellow), DNA (blue,) and GFP-positive transferred hematopoietic stem cells. Immunofluorescence images showing CD3+ T-cell localization in (E) control and (F) adoptive cellular therapy-treated gliomas. (G) Gene expression analysis of T-cell-associated genes in regions of interest from control and adoptive cellular therapy-treated gliomas. Statistical analysis was performed using the 2-way ANOVA test. *P ≤ .05. **P ≤ .01.***P ≤ .001.****P ≤ .0001; error bars: mean ± standard deviation. Abbreviations: ANOVA, analysis of variance; HSCs, hematopoietic stem cells.

It has been previously published that the therapeutic effect of our platform relies upon the transfer of tumor-reactive lymphocytes after myeloablation.^15-17^ Here we characterized the impact of myeloablation with ACT on lymphoid reconstitution in the TME and in secondary lymphoid organs. Twenty-eight days after ACT, tumors and secondary lymphoid organs were harvested, and CD4+ and CD8+ T-cells were quantified via flow cytometry. *Glioma*: We observed that CD4+ T-cells and CD8+ T-cell reconstitution was significantly increased after myeloablation with HSCs and ACT (Figure 2A and B). *Spleen*: Additionally, while 9-Gy myeloablation and HSCs significantly reduced CD4+ T-cells in the spleen, groups receiving 9-Gy myeloablation, HSCs, and ACT had CD4+ T-cell counts to levels equivalent to all control groups. CD8+ T-cells were significantly reduced in the spleen after myeloablation and myeloablation with HSCs and ACT compared with controls (Figure 2C). *Cervical lymph nodes*: We observed that the reconstitution of CD4+ T-cells in the cervical lymph nodes was significantly increased in groups that received 9-Gy myeloablation with HSCs and myeloablation with HSCs and ACT relative to tumor control and non-irradiated mice treated with HSCs and ACT (Figure 2D). However, CD8+ T-cell levels in the cervical lymph nodes had no change in reconstitution due to either ACT or 9-Gy myeloablation.

Our whole transcriptomic analysis using GeoMx DSP (Figure 2E-G) revealed that mice receiving 9-Gy myeloablation, HSCs, and ACT significantly increased *CD3g, CD3d,* and *CD3e* expression in the TME relative to 9-Gy myeloablation and HSC-treated mice (Figure 2G). Quantitative transcriptomic analysis corroborated these findings and were visualized using heatmap analysis identifying differentially expressed T-cell-associated genes after treatment (Figure 2G). Multiple T-cell-associated transcripts were significantly increased, including *Cd8a* and *Cd4* in mice treated with 9-Gy myeloablation, HSCs, and ACT compared with mice treated with 9-Gy myeloablation and HSCs. Additionally, several genes associated with T-cell activation, including *Il2ra* and *Pdcd1*, were significantly upregulated after 9-Gy myeloablation, HSCs, and ACT compared with 9-Gy myeloablation and HSCs (Figure 2G). Taken together, our observations demonstrate that 9-Gy myeloablation and HSCs significantly decreased MDSC and TAM populations in the TME. However, the combination of 9-Gy myeloablation and ACT led to an increase in pro-inflammatory T-cell signaling and anti-tumor immune activation.

T-cell frequencies in secondary lymphoid organs and glioma tissue after 5-Gy lymphodepletive and 9-Gy myeloablative host conditioning and ACT were also quantified using flow cytometry (Supplementary Figure S4A-F). Both CD4+ and CD8+ T-cell frequencies in glioma tissue were significantly increased after myeloablation, HSCs, and ACT compared with 9-Gy myeloablation and HSCs, 5-Gy alone, and 5-Gy with HSCs and ACT (Supplementary Figure S4E and F). Quantitative comparisons in log2-fold T-cell gene expression changes across treatment groups are expressed in Supplementary Figure S4G. Relative T-cell proliferation after 5-Gy lymphodepletive and 9-Gy myeloablative TBI with HSCs and ACT relative to control groups (Supplementary Figure S7A-C) demonstrated that T-cell proliferation was higher after 9-Gy myeloablative TBI.

### ACT Prevents MDSC Migration

**Figure 3.**
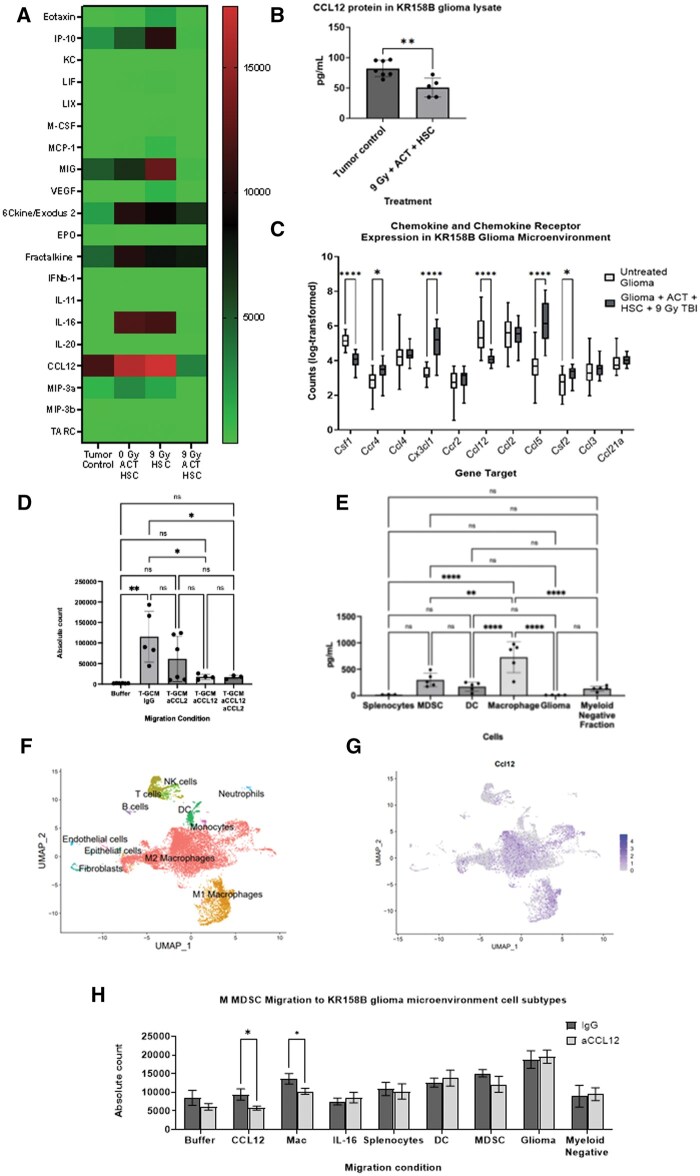
Integrated analysis of chemokine/cytokine protein after 9-Gy myeloablative radiation, HSCs, and adoptive cellular therapy and in vitro migration assays and single-cell RNA sequencing analysis in KR158B glioma. (A) Cytokine and chemokine profiling of glioma protein lysates in the tumor secretome in adoptive cellular therapy–treated gliomas and untreated control. Statistical comparisons were made using the 1-way ANOVA test. (B) CCL12 protein levels in glioma lysates were quantified by ELISA. Statistical analysis was made using the unpaired Student *t* test. (C) Transcriptomic analysis of chemokine ligands and receptors using nanoString digital spatial profiling. Statistical analysis was performed using the 2-way ANOVA test. (D) In vitro trans-well migration assay of MDSCs migrating in response to glioma conditioned media from excised glioma tissue combined with neutralizing antibodies against CCL2 and/or CCL12. Statistical analysis was performed using the 1-way ANOVA test. (E) ELISA CCL12 protein quantification using immune cell–specific isolates from excised glioma tissue. Statistical analysis was performed using the 1-way ANOVA test. (F) Immune cell subset deconvolution from single-cell RNA sequencing analysis from excised glioma tissue. (G) mRNA expression of CCL12 overlayed on immune cell subset deconvolution using single-cell RNA sequencing analysis from excised glioma tissue. (H) In vitro trans-well migration assay of MDSCs migration in response to immune cell-specific isolates combined with either IgG or CCL12 neutralizing antibody. Statistical comparisons conducted using the 2-way ANOVA test. *P ≤ .05. **P ≤ .01. ***P ≤ .001. ****P ≤ .0001; error bars: mean ± standard deviation. Abbreviations: ANOVA, analysis of variance; MDSCs, myeloid-derived suppressor cells.

The above data suggest that while immune reconstitution after ACT occurs in lymphoid organs, there is a failure of MDSC reconstitution in the TME following myeloablation. Thus, we hypothesized that the myeloablation-mediated depletion of MDSCs may be due to decreased cell migration to the TME. To address this hypothesis, we characterized the impact of myeloablation with or without ACT on the secreted proteome in the TME. To do so, KR158B tumor lysates were generated from groups treated with ACT and were analyzed via multiplex protein array. A reduction in CCL12 was found in treatment groups relative to tumor controls (Figure 3A). Quantification by ELISA confirmed that CCL12 concentration was significantly decreased after 9-Gy myeloablation with HSCs and ACT relative to untreated KR158B glioma (Figure 3B). We also evaluated the KR158B TME for transcriptional changes in chemokine/chemokine receptors after 9-Gy myeloablation, HSCs, and ACT relative to 9-Gy myeloablation and HSCs using DSP analysis (GeoMx, as above). We observed that *Ccl12* expression was significantly decreased after myeloablation, HSCs, and ACT (Figure 3C). Like our proteome dataset, *Ccl2* expression was not significantly decreased after treatment, suggesting that CCL2-mediated MDSC migration may not impact MDSC depletion in ACT (Figure 3C). Additionally, *Ccr2* and *Ccr4* expression was measured given that they serve as receptors for *Ccl12* and *Ccl2*. While *Ccr2* expression was not statistically different, *Ccr4* was significantly increased after 9-Gy myeloablation, HSCs, and ACT relative to 9-Gy myeloablation and HSCs (Figure 3C).

Given CCL12’s role within the monocyte chemoattractant protein family, we then hypothesized that loss of CCL12 may be a potential mechanism underlying MDSC depletion in the KR158B TME.^35^ To determine this, we measured the impact of CCL12 neutralization on MDSC migration using KR158B glioma conditioned media (GCM).^22^ CCL2 neutralization in KR158B GCM did not significantly decrease MDSC migration, whereas CCL12 neutralization or combined CCL12/CCL2 neutralization significantly decreased MDSC migration (Figure 3D). While CCL2/CCR2-mediated MDSC migration in KR158B glioma is well-documented, our observations indicate that CCL12 may exert more potent stimulation of cell migration than CCL2 in vitro. We extended these studies to evaluate the impact of CCL12 neutralization using GL261 GCM. Like our findings in KR158B, we observed a significant decrease in MDSC migration following CCL12 neutralization in GL261 GCM in vitro (Supplementary Figure S9D-F).

As CCL12 neutralization in ex vivo KR158B GCM significantly abrogated MDSC migration, we hypothesized that CCL12 may be secreted by non-neoplastic cells in the KR158B TME. However, it is unknown which cell type in the KR158B TME secretes CCL12. To test this, KR158B glioma was isolated after 28 days post-implantation. Magnetic bead isolation was used to isolate MDSCs, DCs, macrophages, glioma cells, and myeloid-negative immune cell populations from this suspension. From this, we identified that CCL12 protein level among macrophages was significantly increased relative to all other cells (Figure 3E). We also repeated this experiment utilizing GL261 and observed consistent findings where CCL12 protein was significantly increased among macrophages relative to all other cell isolates (Supplementary Figure 9A). To validate these results, untreated KR158B glioma tissue was isolated at the same time point, and single-cell RNA sequencing was performed. *Ccl12* RNA was found to be highly expressed in the M2-macrophage and M1-macrophage cluster (Figure 3F and G). To confirm that macrophage-derived CCL12 protein stimulated MDSC migration, induced MDSCs were plated using a trans-well migration assay as previously described with each eluted cell fraction from the TME plated in the lower Boyden chamber with or without neutralizing antibodies against CCL12. We observed that MDSC migration was significantly decreased after neutralization of macrophage-derived CCL12 (Figure 3H). To validate this finding, we also utilized immune cell subtype isolates from GL261 and observed that CCL12 neutralization from GL261-derived TAMs significantly decreased MDSC migration in vitro (Supplementary Figure S9F).

*Supplementary*: We characterized the impact of myeloablation with ACT on chemokine/cytokine protein level in serum. We did not observe significant differences in CCL2 or CCL12 (Supplementary Figure S8A). Additionally, using KR158B tumor control mice, we quantified CCR2 and CCR4 protein levels on MDSCs by flow cytometry as our transcriptomic data identified differences in *Ccr4* expression in the TME. We observed that CCR2 protein level is significantly higher than CCR4 protein level, indicating that CCR2 is the predominant receptor for CCL2 and CCL12 (Supplementary Figure S8B). Next, we sought to determine whether CCL12 stimulated MDSC migration in vitro. We observed that recombinant CCL12 significantly increased MDSC migration in a concentration-dependent manner but was abrogated by CCL12 neutralizing antibodies (Supplementary Figure S8C and D). We next determined the impact of CCL2 and CCL12 neutralization in KR158B GBM (monolayer and ex vivo derived GCM) and found both anti-CCL2 and anti-CCL12 reduced MDSC migration relative to KR158B GCM and IgG treated samples (Supplementary Figure S8E). We also examined the impact of CCL2 and CCL12 neutralization on MDSC migration using GL261 GCM (monolayer and ex vivo derived GCM). While CCL12 and/or CCL2 neutralization did not decrease MDSC migration in monolayer GL261 GCM, neutralization of CCL12 and/or CCL2 in *ex-vivo* GL261 GCM significantly decreased MDSC migration, confirming our results with *ex-vivo* KR158B GCM (Supplementary Figure S9D and E).To determine if CCL2 and CCL12 were being secreted by KR158B tumor cells or non-neoplastic cells in the TME, we evaluated the relative protein abundance of CCL2 and CCL12 in KR158B GBM monolayer (a monolayer of KR158B cells) and ex vivo cultured KR158B (tumor cells implanted in a mice, grown for 28 days, excised, and cultured in vitro for 24 hours). We observed that CCL2 protein was not significantly different between monolayer KR158B GCM and ex vivo GCM (Supplementary Figure S3F and G). However, CCL12 protein was significantly increased in ex vivo KR158B GCM relative to CCL12 protein in monolayer KR158B GCM and significantly increased relative to CCL2 protein in ex vivo KR158B GCM (Supplementary Figure S3F and G). Additionally, we repeated this analysis using GL261 monolayer and ex vivo GCM to measure CCL12 and CCL2 protein. While CCL2 protein was not significantly different between GL261 monolayer and ex vivo GCM, we found that CCL12 protein was significantly increased in GL261 ex vivo GCM compared with monolayer GCM (Supplementary Figure S9B and C), confirming our results in KR158B.

### High Expression of Orthologous Ccl2 in GBM Is Associated With Worse Clinical Outcomes

**Figure 4.**
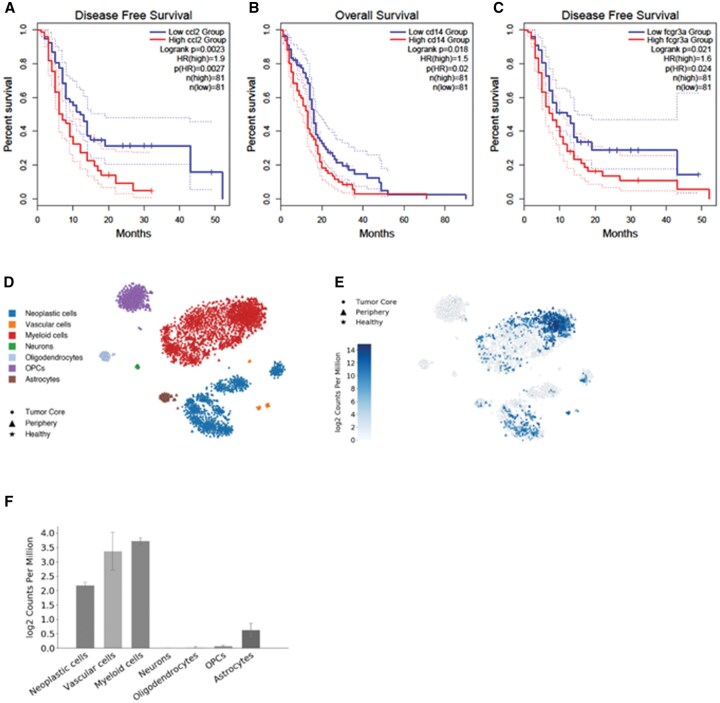
Association of *Ccl2* and macrophage-associated gene expression with clinical outcomes in glioblastoma patients. (A) Kaplan-Meier analysis of disease-free survival based on *Ccl2* expression. (B) Kaplan-Meier analysis of overall survival of *Cd14* expression. (C) Kaplan-Meier analysis of disease-free survival stratified by *Fcgr3a* expression. (D) Deconvolution of single-cell RNA sequencing cell subsets in the glioma microenvironment of GBM patients. (E) Single-cell RNA sequencing deconvolution of *Ccl2* expression among cell clusters from GBM patients. (F) Deconvoluted quantification of *Ccl2* expression from cell clusters identified from GBM patient samples. Statistical comparisons made using the log-rank test with *P* values presented on the survival graphs along with hazard ratios and *P* values for hazard ratios.

Given that *Ccl12* expression is upregulated in the TME, but significantly reduced after ACT, we investigated the association between *Ccl12*’s human ortholog, *Ccl2*, and clinical outcomes among GBM patients. Utilizing TCGA along with analysis from gene expression profiling interactive analysis 2 (GEPIA 2) software, we analyzed gene expression of *Ccl2* among GBM patients. We selected the TCGA GBM cohort and then stratified survival outcomes based on a median cut-off of *Ccl2* expression. This analysis revealed that high *Ccl2* expression was associated with significantly decreased disease-free survival and a hazard ratio of 1.9 (*P* = .0023 by the log-rank test in Kaplan-Meier survival analysis and *P*(HR) = .0027; Figure 4A). This indicates that patients harboring high expression of *Ccl2* have a 90% increased risk of death relative to patients that harbor low *Ccl2* expression.

Given that our ACT platform depletes TAMs from the TME, we investigated associations between the expression of TAM-associated genes, *Cd14* and *Fcgr3a*, and clinical outcomes of GBM patients. Using median expression cut-offs, we identified that high expression (>50%) of *Cd14* was associated with significantly worse overall survival (*P* = .018 by log-rank test) and high expression of *Fcgr3a* significantly decreased progression-free survival (Figure 4B and C) (*P* = .021 by log-rank test). Hazard ratios for *Cd14* and *Fcgr3a* were 1.5 and 1.6, respectively (*P*(HR) = .02, *P*(HR) = .024, respectively), indicating there is a 50% to 60% increased risk of death among patients with high expression of macrophage-associated genes.

To determine cell-specific expression of *Ccl2* in human GBM, we leveraged a publicly available single-cell transcriptomic dataset from human GBM samples.^36^ We observed that *Ccl2* expression was identified among neoplastic cells, vascular cells, and myeloid cells (Figure 4D-F). The highest level of *Ccl2* expression was observed within the vascular and myeloid cell clusters in the TME. This analysis validated our previous results from mice, reinforcing a conserved immunological feature of the HGGs: high expression of *Ccl12* within TAMs.

## Discussion

Treatment of glioma-bearing mice with our novel ACT enabled immune reconstitution in lymphoid organs, consistent with prior reports that transferred HSCs efficiently recover after myeloablative conditioning.^37^ However, MDSCs, which are expanded in the bone marrow and TME of glioma-bearing mice and GBM patients, failed to reconstitute within the TME after myeloablation with TBI. Although our use of TBI is not clinically accurate, regimens involving lymphodepletive chemotherapy in patients produce similar immunologic effects as our platform, exerting severe depletion on inhibitory myeloid cells and regulatory T cells in solid tumors.^15^^,^^37-45^ Thus, our platform models a central observation that host conditioning, through radiation or chemotherapy, augments antitumor immunity. Interestingly, in patients who received preparative lymphodepletion with low or high dose TBI, response rates were increased and observed in all cancer sites, including the brain.^39^ Consequently, the rationale for host conditioning is well founded across multiple preclinical and clinical studies, demonstrating that preconditioning mediates expansion of transferred tumor-reactive T cells, loss of host inhibitory elements, and significant tumor regression in several models, including brain tumors.^15^^,^^37-45^

Other reports also note that lymphodepletive chemotherapeutic regimens exert similar impacts to myeloid cells in the periphery that we observed in our experiments. Our findings identified peripheral expansion of myeloid cells in lethally irradiated mice. Similarly, an expanded myelopoiesis, including MDSCs, CCR2^+^ macrophages and monocytes, is observed after lymphodepletive chemotherapy administration (cyclophosphamide or gemcitabine) in blood of patients.^46-48^ These studies demonstrate potential concordance between the alterations in peripheral immune compartments caused by chemotherapy-based and radiation-based conditioning. Our work expands on this literature by investigating not only changes to peripheral immunity, but to the TME, where MDSCs fail to reconstitute following myeloablation with TBI.

Additionally, our studies of myeloablation may also be extended to other malignant diseases like primary CNS lymphoma where chemotherapy-based myeloablation is a component of clinical consolidation strategies.^49^^,^^50^

While preparative irradiation and chemotherapy are thought to enhance adoptive immunotherapy in part through depletion of suppressive cells, the selective loss of MDSCs in the TME remains poorly understood. Here, we show that MDSCs persisting in the TME exhibited minimal proliferation and underwent apoptosis, suggesting that radiation imposes a selective pressure on their survival in the TME. Thus, whereas systemic compartments were reconstituted, the TME was profoundly depleted of immunosuppressive myeloid elements, establishing a mechanistic basis for enhanced antitumor immunity in HGG.

We show that 9-Gy myeloablation combined with ACT significantly reprogrammed the glioma immune landscape. Transcriptional profiling revealed reduced expression of genes associated with MDSCs and M2-macrophages, alongside enrichment of signatures linked to M1-macrophage inflammation and effector T-cell responses. These findings extend prior work showing that HSCs, a component of ACT, differentiate to antigen-presenting cells and promote T-cell infiltration and activation via interferon-gamma.^15-19^ Our results are also consistent with broader evidence that loss of immunosuppressive signaling enhances antigen presentation and supplements T-cell function against HGGs.^5^^,^^51^ This pattern is shared among other platforms that use CAR T-cells: IL-13Rα2 or an IL-15 modification and found that adoptive transfer of CAR T cells depleted MDSCs, reduced their secretion of immunosuppressive molecules, and significantly increased overall survival against models of HGGs.^52^^,^^53^ Moreover, the transcriptional programs that we observed in glioma-bearing mice treated with ACT closely mirror effector-associated lymphoid signatures observed in patients diagnosed with GBM. Genes related to T-cell activation like *Il2ra* and *Pdcd1* that were previously correlated with improved clinical outcomes in CAR T-cell-treated cohorts were similarly enriched.^54^ Collectively, these data demonstrate that myeloablation with ACT drives a coordinated reprogramming of the glioma TME that converges on molecular signatures of improved outcomes for GBM patients.

The failure of MDSC reconstitution in the TME prompted investigation into migratory cues that influence their chemotaxis. Secretome analysis revealed that myeloablation with ACT markedly reduced CCL12 RNA and protein levels. Functional assays confirmed that CCL12 stimulated MDSC migration, with TAMs identified as the major secretory source. Neutralization of CCL12 significantly impaired MDSC migration, establishing CCL12 as a mediator of MDSC infiltration in glioma. These findings align with other chemotactic axes implicated in glioma-associated myeloid recruitment such as CCL2/CCL7/CCR2 signaling, CSF1R signaling, and CXCL12/CXCR4.^22^^,^^35^^,^^55^^,^^56^ Interestingly, CCR2, CCL12’s receptor, redundantly binds other C-C motif chemokines, including CCL2, and strongly induces CCR2+ cell migration.^22^^,^^35^^,^^56^^,^^57^ Although ACT downregulates CCL12, other CCR2 ligands like CCL2 could potentially sustain MDSC migration to the TME. However, we did not observe significant differences in CCL2 protein or RNA expression, indicating that MDSC migration was likely affected by other chemokines. We also observed that CCL12 expression was restricted to macrophages, whereas CCL2 is redundantly expressed by host immune and neoplastic cells in the TME. Future studies should examine co-targeting of CCR2 ligands, using cell-specific ablation to determine their relative potency in stimulating migration, their impact on the TME, and, ultimately, survival outcomes.

A limitation of this study is the use of a single glioma model to evaluate our ACT platform. However, the KR158B model is among the least immunogenic of several murine brain tumor models, and we anticipate that comparable results would be observed across other low-immunogenicity glioma models.^58^ Given that prior published work showed that ACT significantly increased overall survival against GL261 glioma, we also analyzed CCL12 sourcing and function in GL261 glioma.^20^ Our results showed that ex vivo GL261 GCM served as the predominant source of CCL12 compared with monolayer GCM. Furthermore, CCL12 was predominantly derived from TAMs in GL261 tumors and neutralization of TAM-derived CCL12 significantly reduced MDSC migration. Taken together, these data suggest that the secretory source and function of CCL12 in vitro is not model-specific to KR158B, as supported by concordant observations in GL261.

To determine relevance among human GBM, we analyzed single-cell RNA sequencing datasets from both murine and human GBM, which demonstrated conserved expression of CCL12 and its human ortholog, CCL2, within the myeloid niche of the TME. The immune landscape of mouse and human gliomas shared several features, including MDSC and macrophage infiltration, regulatory lymphocytes, and vascular endothelium.^7^^,^^36^ Notably, human CCL2 expression was extended beyond myeloid cells to neoplastic and vascular compartments, highlighting both conserved and divergent biology.^36^ We further stratified gene expression by survival outcomes in GBM patients and found that elevated CCL2/CCL12 expression and macrophage-associated signatures correlated with worse outcomes, underscoring the translational relevance of this pathway. Our observations also align with recent work showing that genetic ablation of *Ccl12* in diffuse midline glioma simultaneously reduced disease-associated microglia, but increased T-cell-recruiting chemokines and activated cytotoxic T cells, and improved survival.^59^ Beyond HGGs, CCL12 has been identified as an MDSC-associated chemokine in invasive bladder cancer, melanoma, lung metastasis, and gastric cancer with evidence suggesting that it promotes MDSC migration.^60-64^ By demonstrating that ACT with myeloablation decreases CCL12 in the TME, our study links this chemokine to the failure of MDSC reconstitution. Future studies should evaluate in vivo targeting of this pathway to determine its impact on the TME and survival outcomes.

In sum, our work demonstrated that myeloablation with ACT reshapes the glioma immune landscape by systemic reconstitution of myeloid and lymphoid cells while selectively depleting MDSCs and macrophages within the TME. This shift is accompanied by reduced immunosuppressive gene signatures, increased cytotoxic T-cell activation, and M1-macrophage polarization. Mechanistically, this effect is driven in part by the loss of CCL12, a potent recruiter of CCR2+ MDSCs, primarily derived from TAMs. In vitro neutralization of macrophage-derived CCL12 abrogated MDSC migration across 2 brain tumor models, positioning the CCL12-CCR2 axis as a potential therapeutic target in glioma. These findings provide a mechanistic rationale for combinatorial immunotherapeutic strategies that limit MDSC trafficking to potentiate antitumor immunity in HGGs.

## Supplementary Material

vdag054_Supplementary_Data

## Data Availability

Data are available on reasonable request and will be shared by the corresponding author.
