# Supplementary material for: Adoptive cellular therapy prevents reconstitution of myeloid-derived suppressor cells in the glioma tumor microenvironment
Source: Neurooncol Adv. 2026 Mar 7;8(1):vdag054. doi: 10.1093/noajnl/vdag054 (PMC13019303; doi:10.1093/noajnl/vdag054)
Supplement: vdag054_Supplementary_Data [file vdag054_supplementary_data.docx]

**Supplementary Table 1**

| **Product Number** | **Fluorophore** | **Marker** |
| --- | --- | --- |
| L34966 (Thermo Fisher) | AQUA | Live/dead |
| 103112 (Biolegend) | BUV396 | CD45 |
| 101205 (Biolegend) | FITC | CD11b |
| 123118 (Biolegend) | APC-Cy7 | F4/80 |
| 127643 (Biolegend) | BV711 | Ly6G |
| 569011 (Fisher Scientific) | BV786 | Ly6C |
| 100713 (Biolegend) | APC-Cy7 | CD8a |
| 100412 (Biolegend) | APC | CD4 |
| 100206 (Biolegend) | PE | CD3 |
| 364108 (Biolegend) | AF-647 | BrdU |
| 640914 (Biolegend) | FITC | Annexin-V |

**Supplementary Table 2**

| **Cell Type** | **Gating Hierarchy** |
| --- | --- |
| PMN-MDSC | Cells -> single cells -> live singlets -> CD45 positive -> CD11b positive ->F4/80 negative -> Ly6C^lo^Ly6G+ |
| M-MDSC | Cells -> single cells -> live singlets -> CD45 positive -> CD11b positive -> F4/80 negative -> Ly6C^hi^Ly6G negative |
| Macrophage | Cells -> single cells -> live singlets -> CD45 positive -> CD11b positive -> F4/80 positive |
| CD4+ T cell | Cells -> single cells -> live singlets -> CD45 positive -> CD3 positive -> CD4 positive |
| CD8+ T cell | Cells -> single cells -> live singlets -> CD45 positive -> CD3 positive -> CD8 positive |

**Supplementary Figure 1**

**
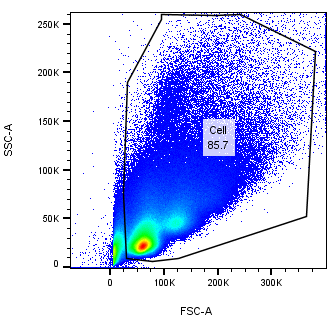

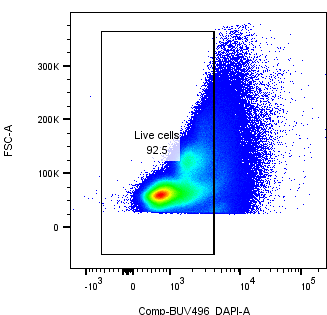

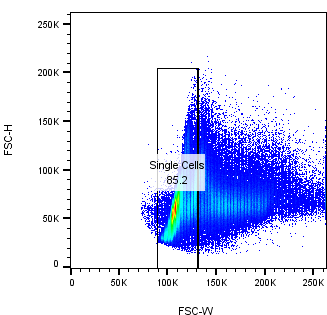

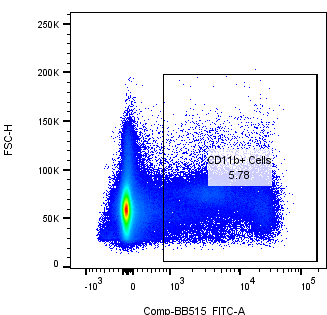

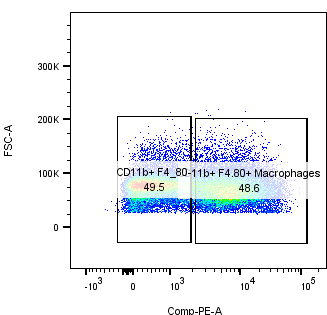

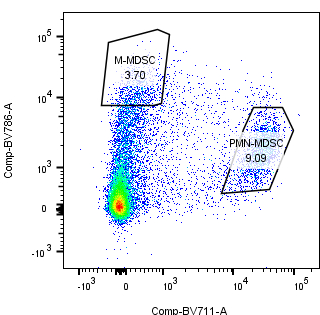

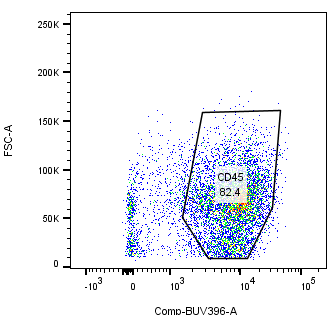
**

**Supplementary Figure 1. Representative flow plots for the myeloid cell gating strategy.** The flow plots above depict the gating strategy that was used to identify myeloid-derived suppressor cells (MDSCs) and macrophages. The overall hierarchy begins with cells then live cells, then live singlets, then CD45+ cells, and then CD11b+ cells. We then gated on both the F4/80 negative and positive populations. F4/80 positive cells were identified as macrophages and F4/80 negative cells then received gating on Ly6C^hi^Ly6G- to indicate monocytic MDSCs and Ly6^lo^Ly6G+ cells to indicate granulocytic MDSCs.

**Supplementary Figure 2**

**
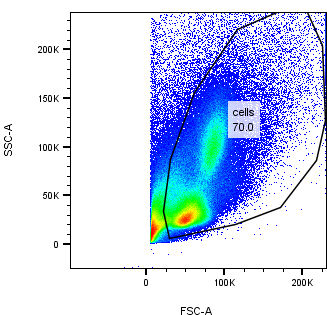

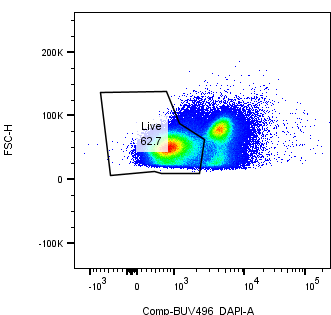

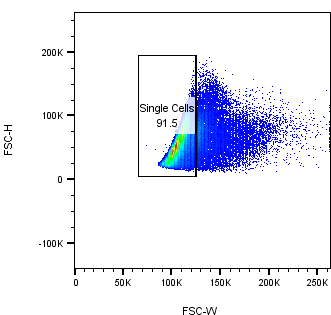

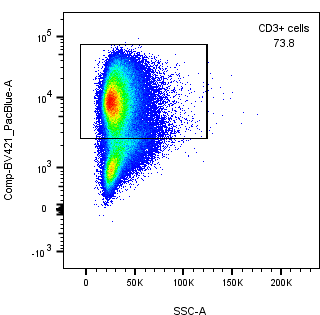

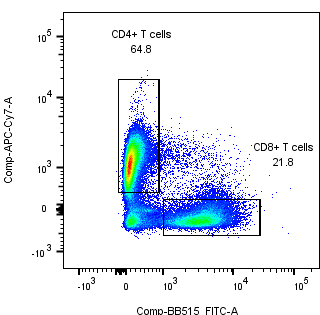

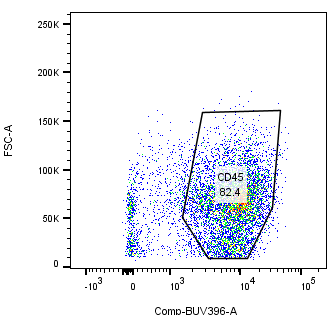
**

**Supplementary Figure 2. Representative flow plots for the lymphoid gating strategy.** The flow plots above depict the gating strategy that was used to identify CD4+ T cells and CD8+ T cells. The overall hierarchy begins with cells then live cells, then live singlets, then CD45+ cells, and then CD3+ cells. We then gated on CD4+ and CD8+ cells. The terminal gates for CD4 and CD8 refer to CD4+ T cells and CD8+ T cells.

**Supplementary Figure 3**

**Supplementary Figure 3. Frequency of myeloid cells across treatment groups with adoptive cellular therapy, HSCs, and total body irradiation in bone marrow, spleen, cervical lymph nodes, and glioma.** a) Frequency of MDSCs and macrophages in bone marrow across treatment groups; statistical analysis was performed using the two-way ANOVA test. b) Frequency of PMN-MDSCs in the spleen across treatment groups; statistical analysis was performed using the one-way ANOVA test. c) Frequency of M-MDSCs in the spleen across treatment groups ; statistical analysis was performed using the one-way ANOVA test. d) Frequency of macrophages in the spleen across treatment groups; statistical analysis was performed using the one-way ANOVA test. e) Frequency of MDSCs in tumor across treatment groups; statistical analysis was performed using the two-way ANOVA test. f) Frequency of macrophages in glioma tissue across treatment groups; statistical analysis was performed using the two-way ANOVA test. g) Frequency of macrophages in cervical lymph nodes across treatment groups; statistical analysis was performed using the one-way ANOVA test. g) Differentially expressed MDSC genes plotted by log2-fold change across treatment groups; statistical analysis was performed using the two-way ANOVA test, using a p-value of less than 0.05 to denote significantly different relationships. i) Differentially expressed M1 and M2 macrophage genes plotted by log2-fold change across treatment groups; statistical analysis was performed using the two-way ANOVA test, using a p-value of less than 0.05 to denote significantly different relationships. *p≤0.05, ** p≤0.01, ***p≤0.001, ****p≤0.0001; error bars: mean +/- standard deviation.

**Supplementary Figure 4**

**Supplementary Figure 4. Frequency of T-cells across treatment groups with adoptive cellular therapy, HSCS, and total body irradiation in spleen, cervical lymph nodes, and glioma.** a) Frequency of CD4+ T cells in the spleen across treatment groups; statistical analysis was performed using the one-way ANOVA test. b) Frequency of CD8+ T cells in the spleen across treatment groups; statistical analysis was performed using the one-way ANOVA test. c) Frequency of CD4+ T cells in the cervical lymph nodes across treatment groups; statistical analysis was performed using the one-way ANOVA test. d) Frequency of CD8+ T cells in cervical lymph nodes across treatment groups; statistical analysis was performed using the one-way ANOVA test. e) Frequency of CD4+ T cells in the glioma tissue across treatment groups; statistical analysis was performed using the one-way ANOVA test. f) Frequency of CD8+ T cells in the glioma tissue across treatment groups; statistical analysis was performed using the one-way ANOVA test. g) Differentially expressed T cell activation genes plotted by log2-fold change across treatment groups; statistical analysis was performed using the two-way ANOVA test, using a p-value of less than 0.05 to denote significantly different relationships. *p≤0.05, ** p≤0.01, ***p≤0.001, ****p≤0.0001; error bars: mean +/- standard deviation.

**Supplementary Figure 5**

**Supplementary Figure 5. Frequency of S-phase+ myeloid cells in treatment groups with adoptive cellular therapy. HSCS, and total body irradiation in bone marrow, spleen, cervical lymph nodes, and glioma.** a) Frequency of S-phase+ MDSCs in bone marrow across treatment groups; statistical analysis was performed using the two-way ANOVA test. b) Frequency of S-phase+ macrophages in bone marrow across treatment groups; statistical analysis was performed using the one-way ANOVA test. c) Frequency of S-phase+ MDSCs in the spleen across treatment groups; statistical analysis was performed using the two-way ANOVA test. d) Frequency of S-phase+ macrophages in the spleen across treatment groups; statistical analysis was performed using the one-way ANOVA test. e) Frequency of S-phase+ MDSCs in the cervical lymph nodes across treatment groups; statistical analysis was performed using the two-way ANOVA test. f) Frequency of S-phase+ macrophages in the cervical lymph nodes across treatment groups; statistical analysis was performed using the one-way ANOVA test. g) Frequency of S-phase+ MDSCs in the glioma tissue across treatment groups; statistical analysis was performed using the two-way ANOVA test. h) Frequency of S-phase+ macrophages in the glioma tissue across treatment groups; statistical analysis was performed using the one-way ANOVA test. *p≤0.05, ** p≤0.01, ***p≤0.001, ****p≤0.0001; error bars: mean +/- standard deviation.

**Supplementary Figure 6**

**Supplementary Figure 6. Frequency of apoptotic and dead MDSCs in treatment groups with adoptive cellular therapy and total body irradiation in spleen and glioma.** a) Frequency of apoptotic PMN-MDSCs in the spleen across treatment groups; statistical analysis was performed using the one-way ANOVA test. b) Frequency of dead PMN-MDSCs in the spleen across treatment groups; statistical analysis was performed using the one-way ANOVA test. c) Frequency of apoptotic M-MDSCs in the spleen across treatment groups; statistical analysis was performed using the one-way ANOVA test. d) Frequency of dead M-MDSCs in the spleen across treatment groups; statistical analysis was performed using the one-way ANOVA test. e) Frequency of apoptotic PMN-MDSCs in the glioma tissue across treatment groups; statistical analysis was performed using the one-way ANOVA test. f) Frequency of dead PMN-MDSCs in the glioma tissue across treatment groups; statistical analysis was performed using the one-way ANOVA test. g) Frequency of apoptotic M-MDSCs in the glioma tissue across treatment groups; statistical analysis was performed using the one-way ANOVA test. h) Frequency of dead M-MDSCs in the glioma tissue across treatment groups; statistical analysis was performed using the one-way ANOVA test. *p≤0.05, ** p≤0.01, ***p≤0.001, ****p≤0.0001; error bars: mean +/- standard deviation.

**Supplementary Figure 7**

**Supplementary Figure 7. Characterization of proliferation states among T lymphocytes in secondary lymphoid organs and glioma after myeloablation and adoptive cellular therapy.** a) Frequencies of S-phase+ CD4+ and CD8+ T cells in the spleen across treatment groups; statistical analysis was performed using the two-way ANOVA test. b) Frequencies of S-phase+ CD4+ and CD8+ T cells in the cervical lymph nodes across treatment groups; statistical analysis was performed using the two-way ANOVA test. c) Frequencies of S-phase+ CD4+ and CD8+ T cells in the glioma tissue across treatment groups; statistical analysis was performed using the two-way ANOVA test. *p≤0.05, ** p≤0.01, ***p≤0.001, ****p≤0.0001; error bars: mean +/- standard deviation.

**Supplementary Figure 8**

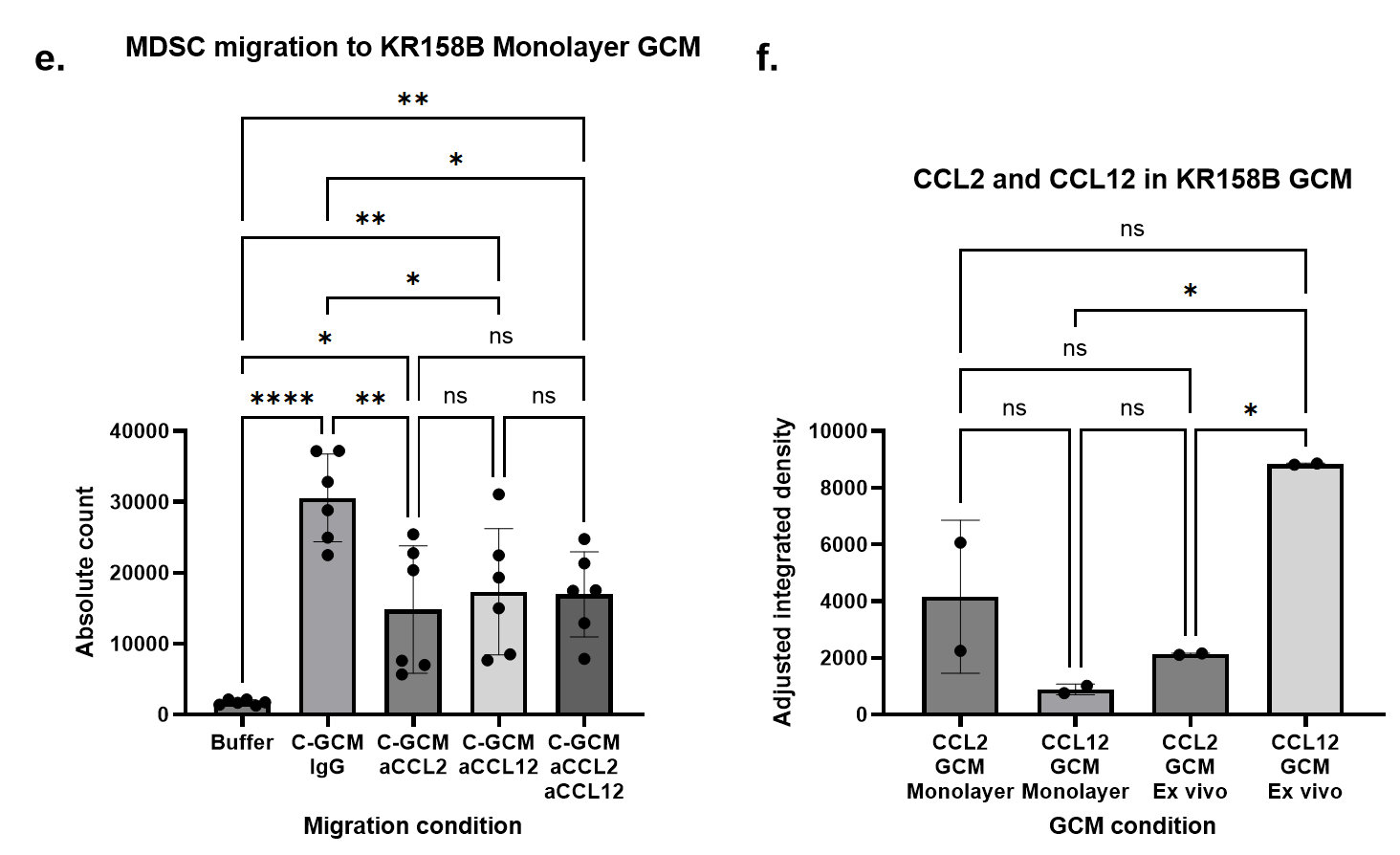


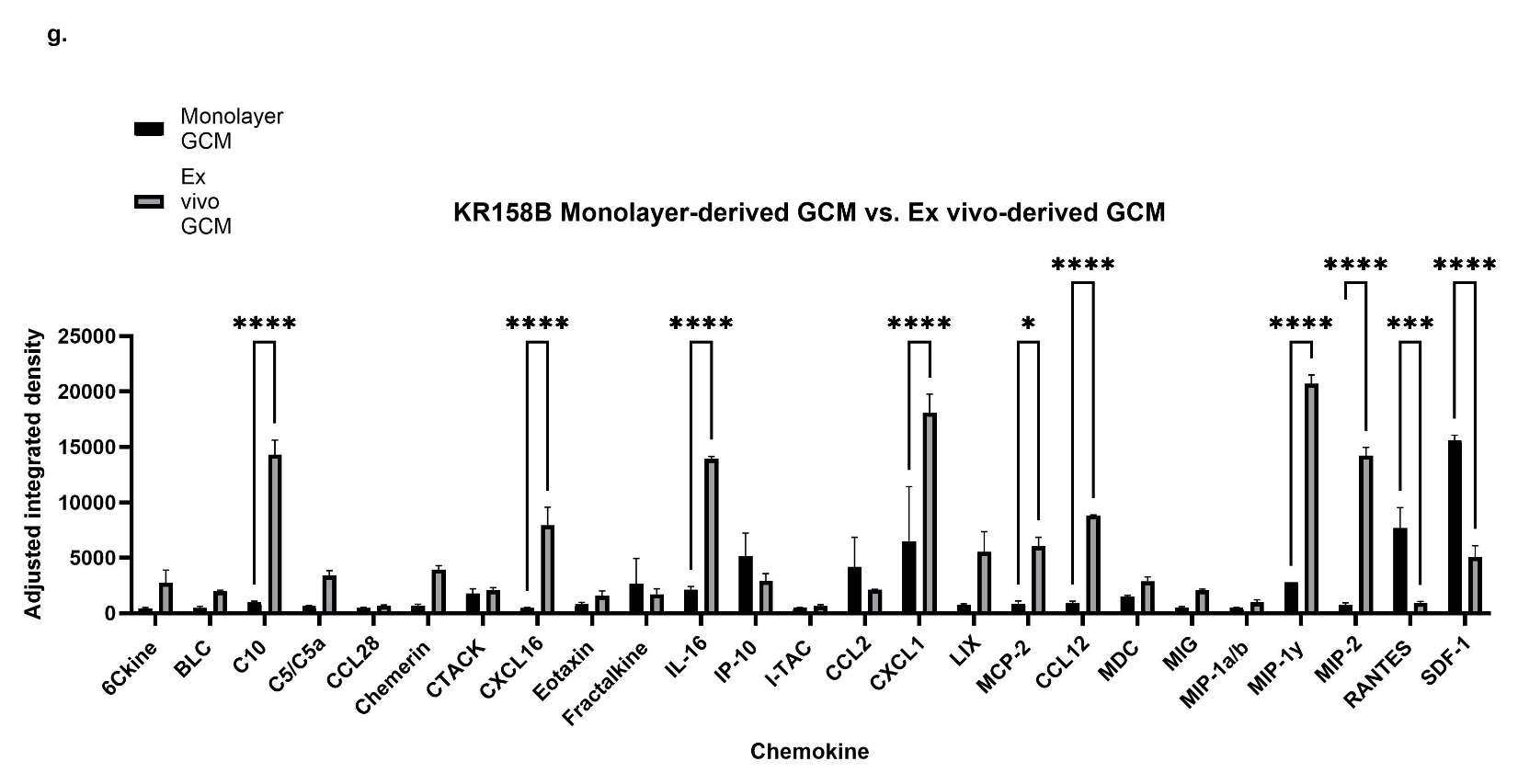


**Supplementary Figure 8. In vitro migration of myeloid-derived suppressor cells in response to recombinant CCL12 and multiplex chemokine array of serum and monolayer and *ex vivo* KR158B glioma conditioned media.** a) Serum collected from mice treated with adoptive cellular therapy was evaluated for chemokines using a murine chemokine proteome profiler. Relative signal intensities were quantified by densitometry and normalized to internal reference spots. b) Quantification of CCR2 and CCR4 on MDSCs from KR158B glioma-bearing mice using flow cytometry; statistical analysis was performed using an unpaired student t-test. c) Trans-well migration assays were performed using MDSCs in the presence of recombinant CCL12. CCL2 served as a positive control and no chemokine (buffer only) and TPO served as a negative control; statistical analysis was performed using the one-way ANOVA test. d) Trans-well migration assay was performed with MDSCs in the presence of recombinant CCL12 with or without neutralizing antibodies against CCL12. CCL2 served as a control along with groups that also received neutralizing antibodies against CCL2; statistical analysis was performed using the one-way ANOVA test. e) Trans-well migration assay was performed using KR158B glioma conditioned media derived from KR158B monolayer culture and conditions were treated with neutralizing antibodies against CCL2, CCL12, or both compared to conditions treated with IgG; statistical analysis was performed using the one-way ANOVA test. f) Conditioned media derived from KR158B monolayer or ex vivo KR158B tumor was analyzed using a murine chemokine proteome profiler array to identify CCL2 and CCL12; statistical analysis was performed using the one-way ANOVA test. g) KR158B glioma conditioned media was analyzed for chemokines using a murine proteome profiler. Relative signal intensities were quantified by densitometry and normalized to internal reference spots. Statistical analysis was performed using a one-way ANOVA; *, p < 0.05; **, p < 0.01; ***, p < 0.001; ****, p < 0.0001. *p≤0.05, ** p≤0.01, ***p≤0.001, ****p≤0.0001; error bars: mean +/- standard deviation.


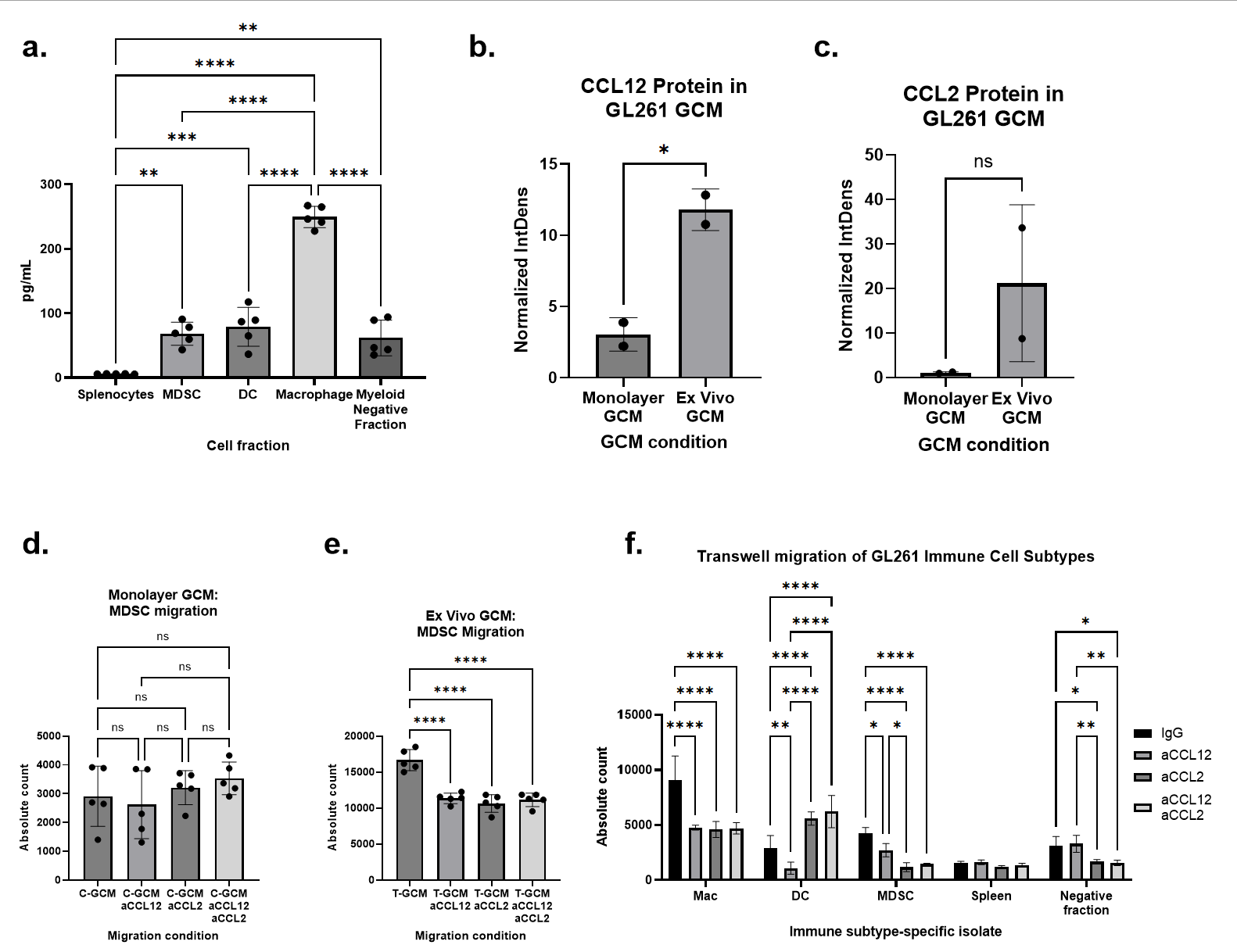


**Supplementary Figure 9. CCL2 and CCL12 protein quantification and MDSC migration using GL261 glioma model.** a) CCL12 protein quantification of MDSC, DC, and macrophage cell fractions from GL261 tumor-bearing mice. b) CCL12 protein quantification in monolayer and ex vivo GCM from GL261. c) CCL2 protein quantification in monolayer and ex vivo GCM. d) MDSC migration using trans-well migration assay containing monolayer GCM with or without neutralizing CCL2 and CCL12 antibodies. e) MDSC migration using trans-well migration assay containing ex vivo GCM with or without neutralizing CCL2 and CCL12 antibodies. f) MDSC migration using trans-well migration assay containing eluted cell fraction from the TME plated in the lower Boyden chamber with or without neutralizing antibodies against CCL2 and CCL12. Statistical analysis was performed using the one-way ANOVA test; *, p < 0.05; **, p < 0.01; ***, p < 0.001; ****, p < 0.0001; error bars +/- standard deviations.
